# Sensorimotor Processing in Elite and Sub-Elite Adolescent Sprinters during Sprint Starts: An Electrophysiological Study

**DOI:** 10.3390/sports12080222

**Published:** 2024-08-15

**Authors:** Yueh-Ling Hsieh, Shiuk-Wen Yen, Chia-Ming Chang, Wei-Chun Li, Nian-Pu Yang, Han-Yu Chen

**Affiliations:** 1 Department of Physical Therapy, Graduate Institute of Rehabilitation Science, China Medical University, Taichung 40402, Taiwan; shiukwenn96@gmail.com (S.-W.Y.); cmchang.cliff@mail.cmu.edu.tw (C.-M.C.); 2 Department of Physical Therapy, Hungkuang University, Taichung 40433, Taiwan; d94428005@sunrise.hk.edu.tw; 3 School of Medicine, National Defense Medical Center, Taipei 114201, Taiwan; cmuhsieh@gmail.com

**Keywords:** elite adolescent sprinter, sensorimotor processing, auditory evoked potentials, sprint start, EEG, EMG, audiomotor reaction time

## Abstract

Most studies on sprint performance have focused on kinematics and kinetics of the musculoskeletal system for adults, with little research on the central sensorimotor transmission and processes, especially for adolescent sprinters. This study aimed to determine whether differences in the integrity of the central auditory system and audiomotor transmissions between the elite and sub-elite adolescent sprinters may affect their performance in the 100 m time. Twenty-nine adolescent junior high school students, including elite national-class and sub-elite regional-class athletes, were assessed. Visual and auditory evoked potentials (VEP and AEP) as well as electroencephalography (EEG) and electromyography (EMG) were recorded and analyzed during a sprint start. The electrophysiological results clearly reveal differences in central auditory transmission between elite and sub-elite groups, and between sexes. There were significant differences between elite and sub-elite groups, and during a sprint start, the EEG activities for elite female and male athletes showed significant time-dependent differences in peak amplitudes following the three auditory cues (ready, set, and gunshot). These findings can provide coaches with a more comprehensive consideration for sports-specific selection based on the athletes’ individual conditions, e.g., sensorimotor neuroplastic training for providing precise and efficient training methods to improve young sprinters’ performance.

## 1. Introduction

Sprint reaction time to initiating movement in a short running race is critical for a sprinter’s performance and may determine the outcome of the race [1]. The sprint start response is a multi-phase process initiated by the perception of the gunshot. Following this, central processing involving transmission, sensorimotor conversion, triggers the onset of muscle activation and force generation [2]. The duration of each phase significantly influences the sprinter’s reaction time [3]. Sensorimotor processing in the brain may thus play an important role during a sprint start.

The adolescent athlete’s central sensorimotor processing differs from that of an adult athlete, in addition to the differences in physical fitness and muscle mechanics properties related to sports skills [4,5]. However, most research has focused on biomechanics of the peripheral movement system in adult sprinters [2,6,7,8,9]. There is little information on central sensorimotor transmission and processing and its correlation to performance in adolescent sprinters.

The sound of a gunshot is the primary cue triggering the start of a sprinter’s reaction process. After hearing the gunshot, the rates at which sensory stimuli are processed and translated into motor reactions in the brain that are involved in movement preparation and execution may affect the overall sprint start reaction time [10]. Several studies have demonstrated that the electrophysiological responses of the central nervous system to auditory and visual stimuli can be evaluated by auditory (AEP) and visual (VEP) evoked potential, and that simple reaction time can be used to evaluate the neural activity in sensorimotor perception and transformation in athletes’ cerebral cortex [5,11]. Significant differences were noted between volleyball players and the sedentary participants in N145 and P100 waveforms of VEP [12]. Elite badminton players exhibited significantly faster visual reaction times compared to non-elite players. However, no significant difference in auditory reaction time was found between the two groups [13]. Other study has also shown that skilled badminton players had earlier EMG onset times accompanied by a faster visuomotor reaction time as compared with nonathletes [14]. Therefore, it can be hypothesized that elite athletes may have different sensorimotor processing capabilities compared to non-athletes. Reaction training is critical because the physical fitness and cognitive flexibility of young athletes their competence depends on it [15]. It is thus worth exploring to what extent the sensorimotor processing between central auditory and motor systems during a sprint start may determine sprint performance of elite adolescent sprinters. That is, when an athlete hears the sound of a gun and starts to sprint forward, will the sensory and motor transmission and transformation affect their sprint performance, especially for the starting reaction time? In addition, are there differences between elite and sub-elite adolescents in this auditory-to-motor transmission process?

This study investigated differences in sensorimotor processing between elite and sub-elite adolescent sprinters by electrophysiological studies, including the AEP, VEP, brain activity, and audiomotor reaction time during a sprint start. Our key hypothesis proposes that significant differences of audiomotor processing between elite and sub-elite adolescent sprinters could be found by electrophysiological studies.

## 2. Materials and Methods

### 2.1. Participants

Twenty-nine junior high school students (16 males and 13 females, from 13 to 16 years old) participated. Based upon an a priori power sample size calculation, a total of 29 participants were used with a power of 0.99 with effect size of 1.50 and significance level (α) of 0.05 to detect a significant difference between elite and sub-elite groups (G*Power, version 3.1.9.7). Individuals with auditory or visual dysfunction, central nervous system disease, upper or lower limb surgery, musculoskeletal system disease, cardiovascular disease, and those taking any medications affecting their motor and cognitive performance were excluded. Participants and their parents were informed of the requirements and potential risks and benefits of participating in this study, and participants were asked to not have injuries at the time of testing. Written informed consent was obtained from all participants, as well as parental consent. Ethical approval for this study was obtained from the Research Ethics Committee of the China Medical University Institutional Review Board (reference no. CRREC-109-195; NCT04859153) in accordance with the Declaration of Helsinki and the Guidelines for Good Clinical Practice.

All included participants were students in athletic physical education courses from Taichung Municipal Shalu Junior High School (Taichung, Taiwan) and had received at least 1 year of regular sprint training. These well-trained adolescent sprinters were further categorized into two groups according to their race performance, as elite and sub-elite groups. The elite group had already participated in several national competitions and their race time was shorter than the mean best 100 m race time of all participants.

### 2.2. Performance Assessment

Athletes performed 100 m sprint trials individually at maximum effort on an official outdoor track after a regular warm-up. Two 100 m trials with a minimum 4 h intervals of complete rest were performed for this assessment (e.g., 2:00 p.m.—the first trial; 6:00 p.m.—the second trial). Running time was recorded by means of manual timing, and the best 100 m race time was selected as their sprint performance.

### 2.3. Electrophysiological Recordings

All electrophysiological recordings, including evoked potentials, EEG, and EMG, were performed in an electromagnetically isolated room. Any electronic equipment using alternating current was removed or replaced with direct current. Participants were not allowed to respond verbally during the experiment.

#### 2.3.1. Auditory and Visual Evoked Potentials

In this study, AEP and VEP served as tools for reflecting the neuronal activities and sensory processing mechanisms in the brain and primary cortex involved in auditory or visual input stimulation in adolescent sprinters. Environmental conditions (temperature, light, silence, and positioning) for recording evoked potentials in the booth were adequate to maintain the individuals’ comfort, without allowing for them to fall asleep. After careful skin cleansing, the electrodes were placed with adequate electrolytic paste. The exam was started when the base line of the EEG was stable, without interference. A 4-channel Neuro-MEP amplifier (Neuro-MEP, Model no.: 03910508, Neurosoft, Ivanovo, Russia) and an auditory–visual stimulator (Neuro-MEP, S/N 03550608, Neurosoft, Ivanovo, Russia) were used for recording AEPs and VEPs to investigate differences of auditory and visual transmissions between elite and sub-elite groups. AEPs were obtained, including short-latency AEP (SAEP), mid-latency AEP (MAEP), and long-latency AEP (LAEP). Click-type auditory stimulation was given to one ear selected randomly with 80 dB sound intensity, 1 Hz stimulus frequency, and a 0.1 ms stimulation duration. The electrodes were placed at Cz (an active electrode), A1 or A2 (a reference electrode, ipsilateral to the earphone), and Fpz (a ground electrode). Parameters for SAEP recordings were set at a low pass filter of 2000 Hz, a high pass filter at 100 Hz, a stimulus frequency of 10 Hz, and with 2000 click stimulations in a trial. For MAEP recordings, the low pass filter was set at 300 Hz while the high pass filter was at 10 Hz with a stimulus frequency of 5 Hz and with 500 click stimulations; the LAEP recordings were set at a low pass filter of 50 Hz and a high pass filter of 1 Hz while the number of trials was set at 200 click stimulations in a trial. Peak latencies of the SAEP (waves I through V), MAEP (Peak P0, Na, Pa, Nb, and Pb), and LAEP (Peak N1, N2, P2, and P3) were obtained for each participant.

VEP testing is performed at usual ambient light levels during the daytime. The participants sat in a comfortable chair, relaxed their body as much as possible, kept a consistent distance of 60 cm from the screen, and then fixed the head on an adjustable chin rest to ensure that their eyes were level with the center of the screen. Eye position should be monitored throughout the test, focusing on the screen and minimizing head movements and blinking. The test should be suspended when the participant’s gaze or attention is distracted. Visual stimulation was achieved with a checkerboard pattern generated on the monitor using VEP software (Neuro-MEP, S/N 03550608, Neurosoft, Ivanovo, Russia), which consisted of black and white checks whose phase was reversed at a fixed rate of two reversals per second. All pattern-reversal visual stimuli were presented binocularly. The parameters for VEP recordings were set at a low pass filter of 50 Hz, a high pass filter of 10 Hz, there were 200 trials was 200, and the stimulus frequency was 1 Hz. Electrodes were placed at the Oz (active), Fz (reference), and Cz as the ground electrode. Peak latencies of the VEP (N75, P100, and N145 peaks) were obtained for each participant.

#### 2.3.2. Brain Activities and Audiomotor Reaction Time during Sprint Start

To assess central sensorimotor processing during movement preparation including stimulus detection of three start commands, neurotransmission and brain responsiveness, brain activities, and audiomotor reaction time, were primarily collected by EEG and EMG.

**Figure 1:**
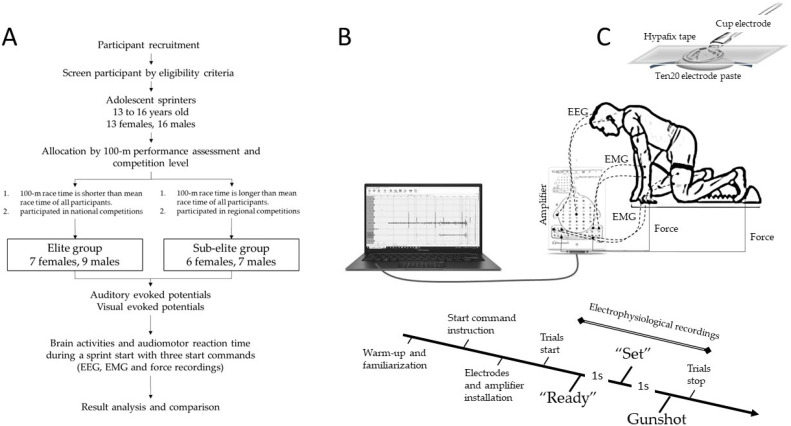
The experimental design (**A**), setup of electrophysiological recordings (**B**), and the secure affixation of EEG electrodes (**C**). A simulated view of the experimental setup when a participant is in ready position with their both hands on the ground and legs on the start blocks. The participant received the three commands, “Ready”, “Set”, and then “Go” (gunshot cue) as simulation with sprint starts. The waveforms of EEG, EMG, and force were recorded when the participant was hearing three commands of a sprint start and performing a sprint start. The connections of EEG, EMG, and force electrodes and recorder amplifier are shown.

All participants had competed at various levels from regional or national competition and were familiar with the use of starting blocks. Before the trials, participants were informed that they would receive the three commands, “Ready”, “Set”, and then a gunshot cue, as simulation for the sprint starts, and that each command was at a 1 sec interval. After a warm-up and familiarization, each participant performed a competitive sprint start at the gunshot sound. Three starts were performed, with 15 min of rest between each trial. The waveforms of EEG, EMG, and force (Neuron-Spectrum-4/P, Model no.: 04900708, Neurosoft, Ivanovo, Russia) were recorded when the participant was hearing three commands for a sprint start and performing the start. Before mounting the electrodes, both alcohol pad and abrasive prep gel (Nuprep Skin Prep Gel, Weaver and Company, Aurora, CO, USA) were used to reduce the impedances of the marked scalp skin where the electrodes were applied. Standard cup electrodes (1.5 m, #TE/C32-634, Technomad, Deerfield, MA, USA) were secured by using electrode paste (Ten20, Weaver and Company, Aurora, CO, USA) and adhesive tape (Hypafix tape, BSN Medical, Luxembourg, Germany). The EEG electrodes were placed at Cz, A1 (reference), and Fpz (ground) for collecting the N1, N2, P2, and P3 waveforms at the period after the sounds of Ready, Set, and gunshot. The parameters of EEG were set at a sampling rate of 5000 Hz, a low pass filter of 35 Hz, and a high pass filter at 0.5 Hz. The EMG activities of the rectus femoris of the rear leg and flexor carpi ulnaris muscles ipsilateral to the rear leg were recorded telemetrically using surface electrodes (LEAD2026S0, LEAD1526S0, Spes Medica S.p.A, Genova, Italy). Parameters of the EMG were a sampling rate of 5000 Hz, a low pass filter of 150 Hz, and a high pass filter of 10 Hz. Force in the rear foot footplate of a standard set of starting blocks and in the ground where the fingers touched and supported were measured with force sensitive resistors (Sensing area: 1.75″ × 1.75″, SEN-09376 (B3-8), SparkFun Electronics, Boulder, CO, USA) to obtain the force signal produced after the gunshot. The experimental design and procedures are shown in Figure 1.

### 2.4. Data Processing

A reaction time task (total reaction time, TRT) can be divided into premotor time (PMT) and motor time (MT), as previously described [16]. PMT is defined by the time interval from the gunshot signal to the initial EMG activation of muscles, while MT is the elapsed time interval between the first change in EMG activity and the onset of exertion (force production). Data acquisition and processing of audiomotor reaction time was performed through a custom-developed computer program (Matlab, R2021b, MathWorks, Natick, MA, USA). The rectified EMG signal was filtered with a cut-off frequency of 35 Hz to obtain the linear envelope. The baseline EMG amplitude was averaged from the 5000 signals during the period from when a participant received the start cue of “Set” before hearing the gunshot. The time elapsed between the auditory signal and the detection of a 5% change relative to the maximum force applied against the block was determined as the TRT. PMT was determined as the time elapsed between the gunshot signal and the detection of a 2× standard deviation (SD) change relative to baseline in the EMG envelope, while MT as the time elapsed between the occurrence of a 2× SD change relative to baseline in the EMG envelope and a 5% change relative to the maximum force applied against the block. The start commands, “Ready”, “Set”, and the gunshot, induced EEG alterations in peak latencies of N1, N2, P2, and P3, as obtained during the PMT period of the sprint start.

### 2.5. Statistical Analysis

The data are presented as means ± SD. Normal distribution of the variables was confirmed by histogram charts and the Shapiro–Wilk distribution. Student *t*-test was used to compare variables in the two independent groups. Two-way analysis of variance (ANOVA) was performed to analyze the effects of the group and sex on each variable. A mixed model two-way analysis of variance was used to analyze the effects of time (within subject factors), groups (between subject factors), and sex (between subject factors) for exploring the differences of serial start commands on the EEG outcomes. Relationships between latencies in electrophysiological studies and 100 m best race time (performance) were assessed by Pearson’s correlation coefficients. Data were analyzed using SPSS statistical software package (version 22; SPSS, Inc., Chicago, IL, USA) and the level of significance was set at alpha = 0.05.

## 3. Results

### 3.1. Demographic Characteristics

All data showed normality of variance. The participant’s characteristics and 100 m race time are described in Table 1. The participants’ mean 100 m race times of 11.93 sec for males and 13.16 sec for females served as the cut-point values for grouping into elite and sub-elite groups. Two-way ANOVA revealed a sex effect in height, weight, BMI, and best race time (*p* < 0.05, Table 1) without sex–group interaction (*p* > 0.05). The mean race times in the elite group (n = 16) were significantly shorter in 9 males and 7 females when compared with those in sub-elite group (n = 13; 7 males and 6 females). According to the competition records, adolescent sprinters in the elite group had more experience in national-class competitions and those in the sub-elite group had more experience in regional-class competitions.

### 3.2. Variation of Auditory and Visual Evoked Potentials

**Figure 2:**
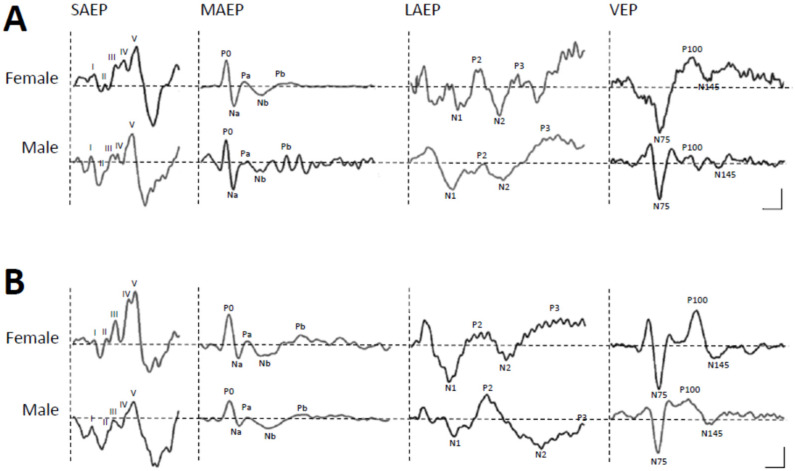
Representative plots of the auditory evoked potentials (AEP) including short-latency AEP (SAEP), mid-latency AEP (MAEP), and long-latency AEP (LAEP), as well as visual evoked potentials (VEP) waveforms recorded from the elite (**A**) and sub-elite groups (**B**). The indicative scale (Sensitivity/Sweep) for SAEP is 2.5 μV/2 ms, for MAEP is 5 μV/10 ms, for LAEP is 4 μV/50 ms, and for VEP is 4 μV/50 ms.

The results of AEP and VEP in latency measurements are described in Table 2 and Figure 2. Of the AEP measures, Wave III, IV, and V in SAEP, as well as P0 and Na in MAEP, exhibited significantly shorter latencies for females than for males (between-sex, *p* < 0.05, Table 2). When calculated between-group, significant differences were found only in Wave IV in SAEP, P0 in MAEP, and P3 in LAEP between two groups (*p* < 0.05), while most of the group differences did not achieve significance in AEP peak latencies. Moreover, there were no interactions between sex and group in all AEP recordings (all *p* > 0.05). Of the VEP measures, there were no significant differences found in sex, group, and sex–group interactions (all *p* > 0.05, Table 2). The peak latencies of SAEP and MAEP seem longer in male sprinters than in females. In the elite group, male sprinters’ Wave III and IV are significantly longer than female sprinters (both *p* < 0.05, Wave III, d = 1.23; Wave IV, d = 1.69). In the sub-elite group, male sprinters’ Wave V, Na, and Pa were clearly longer than those of female sprinters (all *p* < 0.05, Wave V, d = 1.65; Na, d = 1.42; Pa, d = 1.65; Table 2). There were significant group differences between elite and sub-elite groups in SAEP wave IV of male sprinters (*p* < 0.05, d = 1.55), as well as in LAEP N2 of female sprinters (*p* < 0.05, d = 0.25, Table 2).

### 3.3. Alternations of Brain Activities Following Three Commands during a Sprint Start

**Figure 3:**
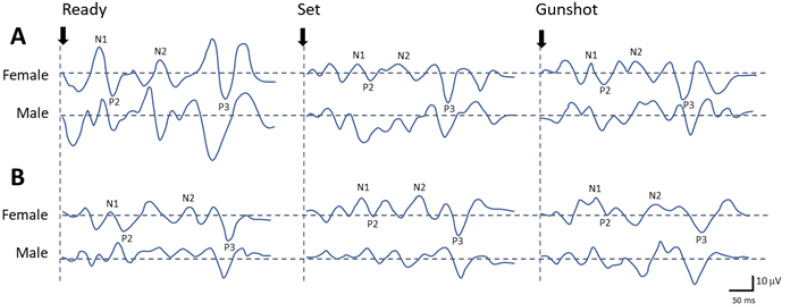
Representative plots of serial changes of EEG waveforms after Ready, Set, and gunshot cues recorded from the elite (**A**) and sub-elite groups (**B**). The indicative scale is 10 μV/50 ms.

Figure 3 shows the alternation of brain activities after Ready, Set, and gunshot (Go) cues recorded by EEG during a sprint start. The EEG responses usually presented two positive peaks, i.e., P2, and P3, and two negative peaks, i.e., N1 and N2 (Figure 3). In the P2 wave, there was a main effect of time (df = 2, F = 9.96, *p* < 0.05) and a main effect of group (df = 1, F = 4.62, *p* < 0.05) found in its amplitude, but a significant interaction between time and group (df = 2, F = 4.48, *p* < 0.05) was found in its latency. The mixed ANOVA with the amplitude of N2 revealed a significant interaction between time and group (df = 2, F = 3.33, *p* < 0.05). In the P3 wave, there were main effects of time (df = 2, F = 9.96, *p* < 0.05) in its latency and amplitude but only a significant interaction among time, group, and sex (df = 2, F = 3.66, *p* < 0.05) was found in its amplitude. As can be seen, the sub-elite groups, showed insignificant changes in amplitudes and latencies in all peaks evoked after three commands of Ready, Set, and gunshot throughout the trial (*p* > 0.05, Table 3). In contrast, both female and male athletes in the elite group showed significant time-dependent differences in the peak amplitudes of N1, P2, and P3 at the three time points (*p* < 0.05). In the elite group, significant decreases in N1 (female, d = 0.97), P2 (male, d = 1.67), and P3 (female, d = 1.75 and male, d = 1.33) peak amplitudes of the gunshot cue-evoked EEG responses were observed in post-hoc comparison with their values evoked by the Ready cue (gunshot vs. Ready, all *p* < 0.05). Pairwise *t*-tests showed a significant difference in the P3 peak amplitude evoked after the Set cue between female and male sprinters of elite groups (*p* < 0.05, d = 2.09), with those in the male sprinters having lower amplitudes than those in the female ones. No significant differences in all peak amplitudes of male sprinters were found between elite and sub-elite groups (all *p* > 0.05, Table 3). The female sprinters in elite and sub-elite groups, however, showed significant differences in peak amplitudes of N1, P2, and P3 evoked individually after Ready, Set, and gunshot cues (all *p* < 0.05, Table 3).

### 3.4. Alternations of Audiomotor Reaction Time Following the Gunshot Cue of a Sprint Start

**Figure 4:**
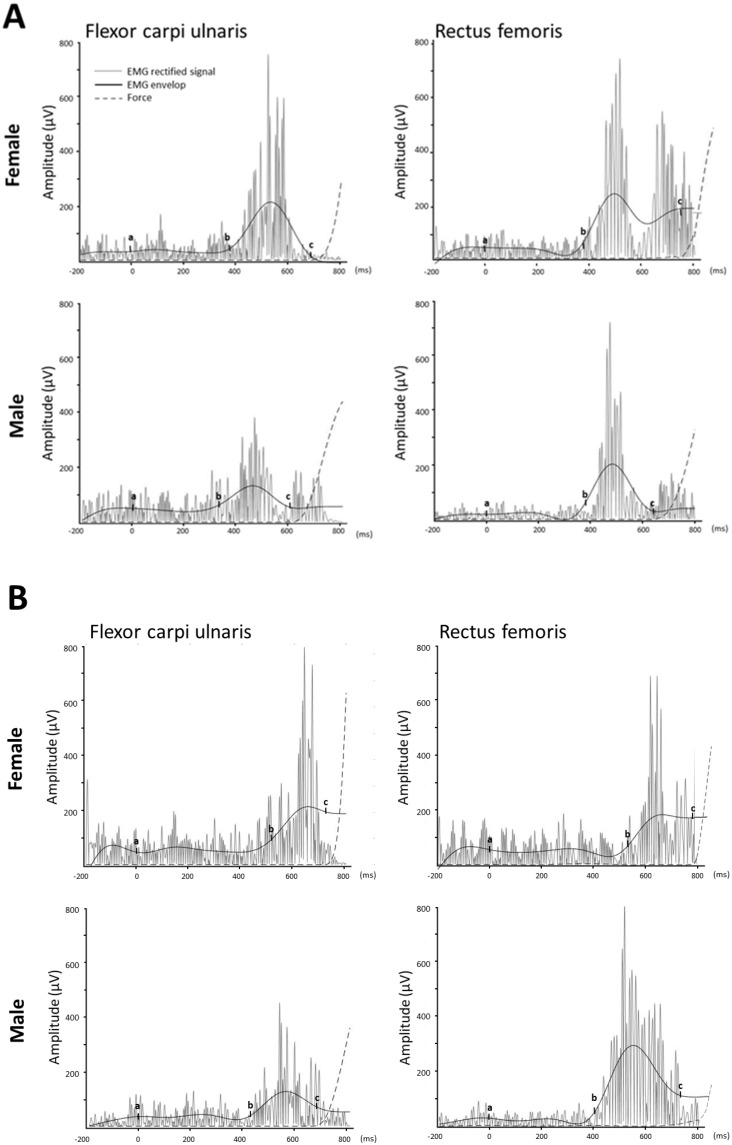
Representative electromyograms (EMG) of flexor carpi ulnaris and rectus femoris muscles of adolescent sprinters in elite (**A**) and sub-elite (**B**) groups during a sprint start trial. Premotor time (RT, a−b interval) was determined as the time elapsed between the gunshot signal (a) and the detection of a 2× standard deviation (SD) change relative to baseline in the EMG envelope (b), while motor time (MT, b–c interval) was the time elapsed between the occurrence of a 2× SD change relative to baseline in the EMG envelope (b) and a 5% change relative to the maximum force (c) applied against the block.

Results of audiomotor reaction time after the gunshot cue of a sprint start containing PMT and MT are shown in Table 4 and Figure 4. There was a main effect on group in the PMT of upper extremities (df = 1, F = 5.51, *p* < 0.05) and a significant interaction between group and sex found in the PMT and TRT of lower extremities (df = 1, PMT: F = 13.96, *p* < 0.05; TRT: F = 6.10, *p* < 0.05). There were no main effects on group, sex, and interaction between group and sex in MT of upper and lower extremities (all *p* > 0.05). Independent *t*-tests showed a significant difference in PMT of upper extremities in the elite group between female and male sprinters (*p* < 0.05, d = 1.67), with male sprinters having longer PMT of upper extremities. But in the sub-elite group, shorter PMT (d = 1.74) and TRT (d = 1.74) of lower extremities were found in male sprinters than in females (Table 4). For female sprinters, the PMT (d = 2.41) and TRT (d = 1.51) of upper and lower extremities were significantly shorter in the elite group than in the sub-elite group (both *p* < 0.05, Table 4).

### 3.5. Correlations of Electrophysiological Measurements and Adolescent Athlete’s Race Time

Without grouping, there were no statistically significant correlations between personal 100 m race time and latencies of electrophysiological studies found in female and male adolescent sprinters (all *p* > 0.05). However, some correlations between race times and latencies were found in female and male sprinters in the elite group (Table 5). Latencies of Waves I and II of SAEP in elite male sprinters, and Wave V in elite females, were significantly and positively correlated to 100 m race times (all *p* < 0.05), implying a positive correlation with performance. But there were no significant negative correlations to race times in latencies of P0 of MAEP and N2 of Set cue evoked potentials for the elite female sprinters (*p* < 0.05, Table 5). The PMT, MT, and TRT had a low to moderate negative correlation with the best race times of elite male sprinters (−0.49 ≥ r ≥ −0.18), except for the PMT of upper extremities, where a very low correlation was found (r = −0.01). However, low to moderate positive correlations with their best race times in the PMT of upper (r = 0.25) and lower extremities (r = 0.48) in elite female adolescent sprinters (Table 5). Nevertheless, the PMT, MT, and TRT of the elite group across extremities and sex did not achieve significant levels correlated with 100 m best race times (*p* > 0.05).

## 4. Discussion

Athlete performance is influenced by complex multifactorial integration and interplay in sport techniques, endurance, strength, and speed. A combination of peripheral and central mechanisms contributes to the speed and motor performance during competition in athletes, although the interaction between these mechanisms remains complex. Animal research suggests that motor speed is regulated by brain regions including the basal ganglia, sensorimotor cortex, and cerebellum [17]. Given this, it is intriguing to explore whether elite adolescent sprinters possess distinct neural characteristics that enable their superior sprint start performance compared to their sub-elite peers. This study used VEP, AEP, EEG, and EMG data from twenty-nine 100 m adolescent sprinters comprising sixteen “elite athletes”, whose best 100 m race time was faster than the average time, and thirteen “sub-elite athletes”, whose fastest time was less than the average. This study reveals the distinct sensorimotor neural profiles of elite adolescent sprinters, highlighting the neural plasticity during adolescence. These findings not only confirm the distinct neural characteristics of these athletes but also underscore the importance of targeted training during this critical developmental period. By utilizing our results, we can design more effective training programs to maximize the potential and performance of elite adolescent athletes, fostering their development into high-performance elite adult sprinters.

Electrophysiologically, VEP components exhibited consistent peak latencies across groups and sexes. In contrast, the early components of AEPs were found to have shorter latency for female adolescent sprinters than for males. Notably, elite adolescent sprinters demonstrated faster early AEP components, suggesting enhanced sensory processing and quicker stimulus response. Early AEP waves (IV and P0) showed significant differences between elite and sub-elite groups, with elite sprinters exhibiting faster wave components associated with better race times. In addition, the observed shorter P3 latency in elite sprinters aligns with previous research suggesting a link between P3 amplitude and cognitive control [18]. Our findings showed that the P3 latency of LAEP (later waves of AEP) was significantly shorter for elite sprinters compared to sub-elite sprinters. These observations, demonstrating enhanced cognitive processing speed in elite athletes, may be crucial for rapid decision-making during competition. Moreover, changes in the main components of latencies and amplitudes of EEG recordings following start cues were accompanied by comparable alterations while participants executed three different sprint start sequences (Ready–Set–Go). Both female and male athletes in the elite group showed significant time-dependent differences in the peak amplitudes of N1, P2, and P3 at the time points of the three cues, demonstrating increased neural activation and synchronization. There were high negative correlations between the Set cue trial N2 latency and best race time values for elite female sprinters, suggesting a critical role of auditory attention in optimizing sprint performance. Elite female adolescent sprinters had significantly shorter PMT in upper and lower extremities than elite male and sub-elite female adolescent sprinters, indicating potential gender-specific neuromuscular advantages. These findings suggest distinct roles for auditory and motor cortex in elite sprint performance, with elite athletes exhibiting neural characteristics adapted for optimal performance.

The electrophysiological responses of the central nervous system to various stimuli can be measured by brain-evoked potentials. Theoretically, almost all sensory modalities can be measured, and the integrity and transmission time of sensations (temperature, pain, etc.) and sense organs (vision, hearing) in the nerve conduction paths can be evaluated. AEPs and VEPs can be used to detect the effects of sound and visual stimulation on brain wave potential changes and can be used to evaluate responses of cerebral cortical activity [19,20]. To date, these response tests have been less commonly used in studies of sports, especially the impact of hearing on sports. A study examining VEPs for a sport-specific visuomotor task in international elite young table tennis athletes found that the amplitude of their VEPs was highly correlated with the visual motor stimulation conditions [21]. Another study, using checkerboard square visual stimulation to evaluate the VEPs of fencers, found significant differences in the wave latency and amplitude of P60, N75, and P100 between fencers and control subjects, especially when processing large field of view stimuli. The shorter wave latency of the players further demonstrates that the visual processing model of fencers differs from that of ordinary people [22]. SAEP analysis revealed significantly shorter III-V interpeak latencies in both male and female tennis and rowing athletes compared to sedentary controls [23]. Therefore, unlike VEP, which appeared to be associated more widely with sports requiring high visual acuity, SAEP appeared to have a broader association with top-level physical activity rather than with specific sensory abilities [23]. The available evidence suggests that waves of SAEP reflect neural activities of sound processing in the auditory pathway originating from brainstem and MAEP reflect intermediate stages of sound processing estimated primarily from mesencephalic structures, midbrain, and subcortical structures [24]. One hypothesis suggests that the observed shorter III-V interpeak latencies in athletes might result from faster synaptic transmission within the superior olivary complex [23]. This current study demonstrates significant differences between elite and sub-elite groups in early waves of AEP (wave IV and P0) and a positive relationship between faster early waves of AEP and best race time in elite adolescent sprinters. Interestingly, between elite and sub-elite sprinters, AEP was more highly correlated with their performance in elite adolescent sprinters than those of VEP. Early AEP potentials reflect sensory processing, largely depending on the stimulus and activities in the lateral lemniscus, inferior colliculus, and the reticular formation [25]. The significant discrepancy found in early AEP potentials may indicate different auditory synaptic transmission occurring in the brainstem, midbrain (including lateral lemniscus, inferior colliculus, and reticular formation), and thalamus–cortical pathways between elite and sub-elite adolescent sprinters. In addition, significant differences on SAEP and MAEP measures were found between females and males, with greater differences on the former. These differences in the waveforms of AEP due to gender were mainly found on the latency, especially the Wave V latency [26], which is consistent with the results of this study. In this study, sex differences were found for Wave III and IV latencies in elite sprinters, but for Waves V, Na, and Pa in sub-elite sprinters. Early waves of AEP in female sprinters were clearly faster than male sprinters. For most SAEP and MAEP measures, the variability in sex differences was greater in sub-elite adolescent sprinters compared to elite ones. Moreover, significant differences between the two groups of adolescent sprinters were noted in early portions (Wave IV and P0) and late portion (P3) of AEP, indicating faster transmission processing in the brainstem and cortical area for elite adolescent sprinters compared to the sub-elite ones. Our findings underscore the critical role of auditory processing in sprint performance, revealing enhanced auditory processing efficiency in elite adolescent sprinters. These results provide compelling evidence for a neural basis of adolescent athletic excellence, emphasizing the importance of auditory perception for rapid decision-making and response initiation in achieving optimal sprint start performance.

To initiate a sprint, athletes must first perceive the auditory cue of the starting gun, then rapidly process this information within the central nervous system before their muscles can react. The sequence of signal processing associated neural transmission is related to the athlete’s overall response time in the sprint start. In a sprint event, after the gunshot, there is a delay of about 3 milliseconds for the sound transmission before it reaches the athlete’s ears [2]. The sound stimulation will first go through stimulation recognition and neuronal coding in the sensory system (cochlear hair cells). Subsequently, the auditory signal is transmitted through the brainstem to the auditory cortex for processing. This information is then integrated in the cerebral cortex before motor commands are sent via efferent nerve fibers originating in the motor cortex. The actual neuromuscular–physiological component (from hair cells, auditory nerve, brain, spinal cord to muscles) of simple auditory reaction time is probably less than 100 ms, possibly due to triggering of the startle reflex through the reticulospinal tract located in the brainstem to activate muscles [27,28]. There has been little research on the sensorimotor component of audiomotor reaction time during sprint starts. Even when considering the shortest possible neural pathway—a spinal reflex via the reticulospinal tract—there is an inherent delay between the motor cortex’s signal and the observable onset of EMG activity [29,30]. The EMG activity of various muscles in sprinting at constant speeds has been documented [31,32]. But there is currently a lack of EEG activity evidence to prove the extent to which the sprint start time is affected by duration of cortical processing in sprinters, and there is even further a lack of relevant research on those aspects in adolescent athletes. Therefore, the EEG activities during a sprint start sequence (Ready–Set–Go) were recorded and investigated up to the end of the first contact after the blocks in this study. In this study, there were no significant variation in amplitudes and latencies in all EEG peaks evoked after three commands of Ready, Set, and gunshot sounds in the sub-elite group. But there were significant deviations found in the peak amplitudes of N1, P2, and P3 among three phases of commands in the elite group. The early waveforms of N1 and N2 in the auditory cerebral cortex evoked potentials largely depend on the neural activities of the auditory cortex, mainly reflecting sensory (auditory) functions, while the later waveforms following P3 mainly record cognitive-related neural activities, reflecting cognitive processing activities [18]. Some researchers claim that sports training and professional experience can lead neuroplastic changes in the human central nervous system and motor cortex for adaptation [33]. It was also shown that that there were significant differences in cortical excitability for athletes compared to non-athletes [34]. It may be that adaptive changes and cognitive processing in the cortical activities are probably critical differences between elite and sub-elite adolescent sprinters. Moreover, we also found there were significant negative correlations to race time in latencies of N2 during set position in the elite female sprinters. The results may be related to anticipatory postural adjustment during set position that must be coordinated by the CNS to achieve desired movement, while also maintaining stability in a preparatory position in anticipation of the dynamics of the start [28]. Our findings reveal distinct EEG patterns between elite and sub-elite athletes, suggesting differences in cortical activation and processing during the sprint start sequence. The observed variations in N1, P2, and P3 amplitudes in elite sprinters indicate enhanced neural efficiency in processing auditory stimuli and preparing for the subsequent motor response. These findings highlight the potential of EEG as a tool to assess cortical readiness and identify neural factors contributing to sprint start performance.

Previous research, primarily focused on biomechanics, has consistently emphasized the critical role of an efficient start in determining sprint race outcomes [33,35,36]. Achieving optimal sprint starts requires a complex interplay of factors, including rapid reaction time, efficient biomechanics, and the integrated function of central nervous system control, physical capabilities, energy systems, and body composition [1,36,37]. The length of reaction time depends on a multifactorial signal transmission process, including the reception of stimulus signals by sensory organs, conversion into neural signals, activation of nerves and neurons for transmission, brain processing, neuromuscular activation, soft tissue compliance, and the selection of external measurements and parameters [38]. Each of these factors needs a relevant processing time that may affect overall audiomotor reaction time. While extensive research has examined the relationship between lower limb reaction time and sprint start performance [28,39], the contributions of upper extremity involvement remain relatively unexplored. A systematic review has established a strong correlation between upper body strength and overall sprint performance [40]. While evidence suggests that arm movements enhance sprint start performance, the specific contributions of arm reaction time and upper body strength remain largely unexplored [41]. To address the limitations of previous research, this study adopts a novel approach by simultaneously recording PMT and MT components of both upper and lower extremities during the sprint start. The results show significantly shorter duration in PMT and TRT of upper and lower extremities in elite female sprinters over sub-elite females. There are also shorter PMTs and TRTs in adolescent female sprinters than those for males in the elite group. However, these findings in contrast to previous results showing that adult male sprinters have significantly shorter reaction times than adult females [42]. In general, these controversies can be explained by different study designs, reaction time measures, and participants’ characteristics; as well as the effect of covariates, such as age; and the different laboratory environment used in electrophysiological studies. Even so, these findings show a weak correlation between reaction components (PMT, MT, and TMT) and 100 m race time for both adolescent males and females, consistent with previous results [42,43]. Male sprinters typically exhibit higher power output, starting velocity, and acceleration due to greater leg muscle strength and explosiveness, enabling them to generate force more rapidly than female sprinters [35]. This could explain the superiority in 100 m race time in adolescent male sprinters over females. Our findings demonstrate significantly shorter reaction times in both upper and lower extremities for elite female sprinters compared to sub-elite females, suggesting a potential advantage in neuromuscular coordination. Additionally, the observed differences between male and female sprinters highlight the complex interplay of factors influencing sprint start performance. These results emphasize the need for a holistic approach to sprint start training, incorporating both upper and lower extremity development.

### Study Limitations

Several methodological limitations are associated with the present study. First, it is reasonable to assume that, due to limitations of the experimental venue, the adolescent sprinters’ start reaction times are longer than for actual performance in a competition situation. However, the results of this experiment still show differences in neuromuscular-physiological components between elite athletes and sub-elite athletes. Accordingly, changes in neuromuscular transmission–conversion processes at the sprint start are underestimated, and the “real” differences between elite and sub-elite groups during the sprint start may be more pronounced under fatigue conditions in an actual competition [44]. The case is similar with the interpretation of P3 waves, which under fatigue conditions would certainly have greater relevance, especially at the sprint start [45,46]. Second, some of these young sprinters surveyed may be more easily distracted during the electro-physiological recordings, which may interrupt the data collection, but this inattention did not necessarily alter the experimental results. Third, although the adolescent sprinters recruited in this study were restricted to junior high school students (13–16 years) and recruited age-matched sub-elite sprinters for comparison, the biological age range is still wider, and the bias caused by age differences cannot be ruled out. Further investigation could be conducted by a cross-sectional study containing a control group with age-matched non-athletes. Additionally, this study may observe talent influences on adolescent athletes under same sprinter training by the same coach by using electrophysiological measurements. But these results may not be extended to older athletes that may have sporting performance advantages due to their favorable anthropometric, physical characteristics and kinematics and experience- or training-related brain plasticity in comparison with adolescent peers [17,47]. However, these findings may be useful because of the homogeneity of the adolescent participants. Although this study also reveals the command-induced audiomotor responses on a sprint start and auditory transmission function, there were limited outcome measurements to help understand the overall differences between elite and sub-elite adolescent sprinters. Using kinematics, biomechanics, biomarker, and neuroimaging approaches, future studies can further evaluate these issues.

## 5. Conclusions

These findings demonstrate that the central neurotransmissions, especially in auditory and sensorimotor systems, differ between elite and sub-elite adolescent sprinters, as well as females and males. The brain activities at the Ready, Set, and Go phases of a sprint start before muscle force develops also differ between elite sprinters and sub-elite ones. Furthermore, the audiomotor reaction time is found to relate to race time following a sprint start. Accordingly, these differences in central sensorimotor processing likely correlate with the sprint performance of adolescent athletes. While research has yet to identify specific structural or functional brain differences between elite and sub-elite adolescent sprinters, the adolescent brain’s capacity for morphological and functional adaptation following motor training is notable. Our findings demonstrated the complexity of the sprint start process including the interplay of cognitive, neuromuscular, and biomechanical components and emphasize the need for a more holistic approach to understanding the factors influencing adolescent sprint performance. Consequently, neuroplasticity training emerges as a potential avenue for enhancing sprint performance. These findings may help coaches develop new training strategies that can modulate neuroplasticity on audiomotor processing during a sprint start for adolescent sprinters.

## Figures and Tables

**Table 1:** Characteristics of the participants in elite and sub-elite groups.

	Elite Group		Sub-Elite Group		^a^ *p* Value		
	Female (n = 7)	Male (n = 9)	Female (n = 6)	Male (n = 7)	Group	Sex	Group × Sex
Age (years)	14.71 ± 1.25	15.00 ± 1.12	14.00 ± 1.10	14.43 ± 0.79	0.13	0.39	0.86
Height (cm)	162.71 ± 6.48	171.40 ± 5.11 ^#^	160.17 ± 8.13	166.57 ± 8.73 ^#^	0.18	<0.01	0.67
Weight (Kg)	48.86 ± 6.44	61.11 ± 4.78 ^#^	47.67 ± 6.68	65.06 ± 7.27 ^#^	0.71	<0.01	0.50
BMI	18.37 ± 1.15	20.78 ± 0.96 ^#^	18.52 ± 1.70	23.17 ± 4.74 ^#^	0.20	<0.01	0.25
100 m race time (s)	12.77 ± 0.11 ^†^	11.50 ± 0.10 ^#,†^	13.62 ± 0.13	12.50 ± 0.17 ^#^	<0.01	<0.01	0.52

^a^: tested by two-way ANOVA; ^#^: *p* < 0.05, significant differences between female and male in each group analyzed by Student’s *t*-test; ^†^: *p* < 0.05, significant differences in each sex between elite and sub-elite groups analyzed by Student’s *t*-test.

**Table 2:** The peak latencies of auditory and visual evoked potentials recorded from adolescent sprinters in elite and sub-elite groups.

		Elite Group		Sub-Elite Group		F Value		
		Female (N = 7)	Male (N = 9)	Female (N = 6)	Male (N = 7)	Group	Sex	Group × Sex
Auditory evoked potentials (AEP)								
Short-latency	Wave I	1.83 ± 0.40	1.96 ± 0.34	1.87 ± 0.29	1.95 ± 0.22	0.01	0.74	0.04
	Wave II	2.90 ± 0.50	2.91 ± 0.40	2.81 ± 0.53	3.06 ± 0.45	0.02	0.55	0.44
	Wave III	3.65 ± 0.56	4.18 ± 0.24 ^#^	3.79 ± 0.34	3.96 ± 0.26	0.09	6.74 ^‡^	1.71
	Wave IV	4.77 ± 0.41	5.48 ± 0.43 ^#^	4.55 ± 0.44	4.85 ± 0.38 ^†^	7.36 ^‡^	10.77 ^‡^	1.68
	Wave V	5.92 ± 0.27	6.19 ± 0.46	5.73 ± 0.17	5.95 ± 0.08 ^#^	3.61	4.74 ^‡^	0.04
Mid-latency	P0	11.63 ± 0.74	12.08 ± 0.64	12.02 ± 0.74	12.84 ± 0.88	4.28 ^‡^	5.18 ^‡^	0.45
	Na	15.92 ± 1.28	17.36 ± 1.51	16.28 ± 0.95	17.61 ± 0.93 ^#^	0.43	9.04 ^‡^	0.01
	Pa	21.36 ± 2.74	21.59 ± 2.31	20.99 ± 0.74	22.27 ± 0.81 ^#^	0.05	1.06	0.52
	Nb	38.13 ± 2.40	38.87 ± 1.15	39.03 ± 1.60	40.13 ± 1.23	3.11	2.23	0.08
	Pb	61.84 ± 9.01	62.08 ± 16.27	60.29 ± 14.52	64.74 ± 15.57	0.01	0.15	5.32 ^‡^
Long-latency	N1	84.16 ± 9.71	79.17 ± 17.95	82.72 ± 15.16	77.31 ± 18.95	0.08	0.74	0.00
	P2	123.18 ± 11.34	108.56 ± 14.76	114.32 ± 10.66	112.35 ± 15.20	0.26	2.73	1.59
	N2	173.57 ± 25.71	154.83 ± 13.78	168.75 ± 9.59 ^†^	167.43 ± 17.81	0.34	2.27	1.71
	P3	218.71 ± 15.26	226.17 ± 22.81	245.50 ± 27.55	248.36 ± 27.83	7.60 ^‡^	0.34	0.07
Visual evoked potential (VEP)								
	N75	84.29 ± 6.66	87.03 ± 5.69	84.43 ± 5.38	85.44 ± 5.45	0.11	0.74	0.16
	P100	109.16 ± 7.64	110.17 ± 6.78	109.56 ± 5.95	111.78 ± 5.77	0.16	0.42	0.06
	N145	146.86 ± 21.61	141.83 ± 16.01	141.00 ± 19.66	144.79 ± 10.68	0.05	0.01	0.46

The values of peak latency are presented as means ± standard deviations. ^#^: *p* < 0.05, significant differences between female and male in each group analyzed by Student’s *t*-test. ^†^: *p* < 0.05, significant differences in each sex between elite and sub-elite groups analyzed by Student’s *t*-test. ^‡^: *p* < 0.05, tested by a two-way analysis of variance.

**Table 3:** The serial alterations of brain activities after ready, set and gunshot cues recorded by EEG during a sprint start in elite and subelite groups.

								Within Subjects Comparison				Between Subjects Comparison		
					Signal Input (Time)			Time	Time × Group	Time × Gender	Time × Gender × Group	Group	Gender	Group × Gender
Wave		Group	Sex	N	Ready	Set	Gunshot	F Value	F Value	F Value	F Value	F Value	F Value	F Value
N1	Latency	Elite	Female	7	75.13 ± 7.37	76.15 ± 5.36	75.75 ± 7.08	0.35	0.09	0.25	0.28	0.00	1.19	1.26
	(ms)		Male	9	75.69 ± 4.80	76.31 ± 6.86	75.15 ± 7.27							
		Subelite	Female	6	75.29 ± 12.97	79.54 ± 6.68	76.75 ± 10.85							
			Male	7	73.89 ± 7.41	73.80 ± 6.29	75.05 ± 8.73							
	Amplitude	Elite	Female	7	18.31 ± 16.15	17.22 ± 12.09	2.64 ± 16.09 *	1.69	0.51	1.55	1.35	0.80	0.06	0.73
	(μV)		Male	9	8.96 ± 24.47	16.02 ± 10.51	5.32 ± 21.20							
		Subelite	Female	6	13.50 ± 26.90 ^†^	8.29 ± 6.53	16.91 ± 16.88							
			Male	7	9.10 ± 20.20	14.17 ± 12.09	9.33 ± 17.51							
P2	Latency	Elite	Female	7	114.89 ± 10.78	117.13 ± 10.79	111.89 ± 13.48	0.26	4.48 ^‡^	0.45	0.81	0.73	1.16	0.02
	(ms)		Male	9	115.98 ± 12.14	112.56 ± 9.83	109.44 ± 12.61							
		Subelite	Female	6	111.08 ± 22.48	108.50 ± 21.20	119.83 ± 16.55							
			Male	7	108.86 ± 11.36	109.75 ± 9.03 ^†^	113.07 ± 15.15							
	Amplitude	Elite	Female	7	46.14 ± 29.66	10.21 ± 16.70 *	27.17 ± 27.57	9.96 ^‡^	1.01	1.70	2.09	4.62 ^‡^	0.52	0.00
	(μV)		Male	9	47.71 ± 12.50	7.45 ± 20.70	16.46 ± 23.41 *							
		Subelite	Female	6	20.18 ± 10.95	21.27 ± 16.76 ^†^	16.25 ± 19.04							
			Male	7	29.20 ± 27.96	21.45 ± 21.77	17.87 ± 14.06							
N2	Latency	Elite	Female	7	156.32 ± 8.10	155.12 ± 11.09	156.63 ± 14.70	1.06	0.42	0.60	1.84	0.01	1.12	1.15
	(ms)		Male	9	158.50 ± 14.25	155.66 ± 15.12	154.03 ± 16.58							
		Subelite	Female	6	163.71 ± 16.76	161.29 ± 20.48	149.50 ± 21.41							
			Male	7	163.06 ± 8.90	162.94 ± 13.11	154.82 ± 12.12							
	Amplitude	Elite	Female	7	20.52 ± 17.48	20.82 ± 25.45	20.52 ± 22.98	1.00	3.33 ^‡^	0.35	1.88	1.87	0.04	0.13
	(μV)		Male	9	22.88 ± 23.61	14.08 ± 16.84	28.21 ± 22.49							
		Subelite	Female	6	37.81 ± 21.74	34.23 ± 22.67	24.14 ± 31.69							
			Male	7	24.08 ± 22.90	49.03 ± 12.22	12.28 ± 19.76							
P3	Latency	Elite	Female	7	215.77 ± 27.29	235.49 ± 22.83	230.06 ± 22.85	4.53 ^‡^	1.24	0.76	1.75	2.71	1.38	1.64
	(ms)		Male	9	221.22 ± 22.20	231.74 ± 21.23	229.80 ± 26.09							
		Subelite	Female	6	244.67 ± 11.88	250.25 ± 24.97	225.83 ± 24.58							
			Male	7	235.56 ± 18.95	242.99 ± 21.30	229.12 ± 18.64							
	Amplitude	Elite	Female	7	60.55 ± 25.50	50.11 ± 10.47	25.54 ± 12.41 *	11.32 ^‡^	0.75	0.23	3.66 ^‡^	0.06	0.00	1.90
	(μV)		Male	9	58.76 ± 28.92	26.81 ± 11.78 *^#^	24.71 ± 21.96 *							
		Subelite	Female	6	54.53 ± 25.00	21.29 ± 11.36	38.95 ± 17.35 ^†^							
			Male	7	57.30 ± 35.80	52.28 ± 15.82	31.13 ± 26.87							

*: tested by Bonferroni compared with Ready sound; ^#^: *p* < 0.05, independent *t*-test between female and male; ^†^: *p* < 0.05, there is significant difference between elite and subelite groups tested by independent *t*-test; ^‡^: *p* < 0.05, tested by a mixed model two-way analysis of variance.

**Table 4:** The audiomotor reaction time of upper and lower extremities recorded from adolescent sprinters in elite and sub-elite groups during a sprint start.

		Elite Group		Sub-Elite Group		Group	Sex	Group × Sex
		Female (n = 7)	Male (n = 9)	Female (n = 6)	Male (n = 7)	F Value	F Value	F Value
Premotor time (ms)	Upper extremity	399.71 ± 29.99	449.96 ± 30.07 ^#^	475.87 ± 33.17 ^†^	457.27 ± 79.55	5.51 ^‡^	0.79	3.75
	Lower extremity	394.48 ± 51.81	434.71 ± 27.2	477.38 ± 45.80 ^†^	402.89 ± 39.43 ^#^	2.77	1.25	13.96 ^‡^
Motor time (ms)	Upper extremity	240.39 ± 38.17	235.95 ± 34.49	255.57 ± 66.43	230.68 ± 32.74	0.09	0.82	0.40
	Lower extremity	246.42 ± 54.51	244.03 ± 88.99	301.27 ± 42.20	256.71 ± 45.30	1.98	0.95	0.77
Total reaction time (ms)	Upper extremity	640.1 ± 54.03	685.90 ± 52.33	731.43 ± 66.65 ^†^	687.95 ± 94.99	3.34	0.00	3.06
	Lower extremity	640.9 ± 80.40	678.74 ± 104.59	778.65 ± 57.36 ^†^	659.60 ± 77.85 ^#^	3.48	1.63	6.10 ^‡^

The data are presented as means ± standard deviations. ^#^: *p* < 0.05, significant differences between female and male in each group analyzed by Student’s *t*-test. ^†^: *p* < 0.05, significant differences in each sex between elite and sub-elite groups analyzed by Student’s *t*-test. ^‡^: *p* < 0.05, tested by a two-way analysis of variance.

**Table 5:** Pearson’s correlation coefficients between latencies of auditory and visual evoked potentials, single trial of the cue-evoked EEG activities, premotor time, motor time, total reaction time of EMG, and personal best 100 m race time in sprinters.

			Elite Female Sprinter		Elite Male Sprinter	
			*r*	*p*	*r*	*p*
Evoked potentials	Short-term auditory evoked potentials	Wave I	−0.24	0.60	0.78 **	0.01
		Wave II	0.08	0.87	0.82 **	0.01
		Wave III	−0.16	0.73	0.36	0.35
		Wave IV	0.52	0.23	0.03	0.94
		Wave V	0.84 *	0.02	0.31	0.41
	Mid-term auditory evoked potentials	P0	−0.78 *	0.04	−0.52	0.16
		Na	−0.56	0.19	0.59	0.09
		Pa	−0.01	0.98	0.62	0.08
		Nb	0.37	0.41	−0.09	0.81
		Pb	−0.59	0.16	−0.03	0.95
	Long-term auditory evoked potentials	N1	−0.27	0.56	0.04	0.91
		P2	−0.21	0.65	0.46	0.21
		N2	0.26	0.57	−0.27	0.48
		P3	−0.57	0.18	−0.50	0.18
	Visual evoked potentials	N75	−0.09	0.84	−0.42	0.27
		P100	−0.47	0.29	−0.36	0.34
		N145	0.06	0.89	−0.08	0.83
Electroencephalography	Ready	N1	−0.64	0.12	−0.27	0.49
		P2	−0.27	0.56	0.19	0.62
		N2	−0.04	0.93	−0.34	0.37
		P3	0.26	0.57	0.36	0.34
	Set	N1	0.49	0.27	0.54	0.13
		P2	−0.24	0.61	−0.16	0.69
		N2	−0.77 *	0.04	−0.15	0.71
		P3	−0.71	0.07	0.59	0.10
	Gunshot	N1	0.31	0.49	0.31	0.41
		P2	0.23	0.62	−0.13	0.74
		N2	0.29	0.53	0.03	0.94
		P3	0.33	0.48	−0.18	0.64
Electromyography	Upper extremity	Premotor time	0.25	0.59	0.01	0.99
		Motor time	−0.08	0.87	−0.49	0.18
		Total motor time	0.09	0.86	−0.32	0.41
	Lower extremity	Premotor time	0.48	0.27	−0.18	0.64
		Motor time	−0.05	0.92	−0.40	0.29
		Total motor time	0.28	0.55	−0.38	0.31

*: *p* < 0.05; **: *p* ≤ 0.01.

## Data Availability

The data used in this study, which includes information about minors, are confidential and cannot be shared due to stringent privacy regulations and ethical considerations. Access to the data is strictly restricted to the research team to protect the identity and well-being of the participants.
